# The Effect of Pulmonary Valve Replacement (PVR) Surgery on Hemodynamics of Patients Who Underwent Repair of Tetralogy of Fallot (TOF)

**DOI:** 10.15171/jcvtr.2015.26

**Published:** 2015

**Authors:** Hamid Bigdelian, Davoud Mardani, Mohsen Sedighi

**Affiliations:** ^1^ Department of Cardiovascular Surgery, Isfahan University of Medical Sciences, Isfahan, Iran; ^2^ Shahid Chamran Heart Center, Isfahan University of Medical Sciences, Isfahan, Iran

**Keywords:** Congenital Surgery, Cardiac Magnetic Resonance, Pulmonary Regurgitation, Prosthetic Valve

## Abstract

*Introduction:* Pulmonary insufficiency (PI) frequently develops in patients who underwent repair of tetralogy of fallot (TOF). The aim of present study was to assess the effect of pulmonary valve replacement (PVR) on hemodynamics of patients who underwent repair of TOF.

*Methods:* This retrospective cohort carried out between July 2010 and October 2012 among consecutive PVRs of 19 patients who underwent TOF surgery. The PVRs was performed using bioprosthetic (n=17) and mechanical (n=2) valves. Our data was collected during follow up visits within 6 to 12 month after PVR.

*Results:* Our results show that PVR significantly decreased right ventricular end-diastolic volume (180.89±13.78 vs. 107.21±12.02 ml/m^2^, *P* < .01), right ventricular end-systolic volume (105.42±15.98 vs. 58.15±11.67 ml/m^2^, *P* < .01), RV mass (47.78±6.20 vs. 30.68±8.95 g/m^2^, *P* < .01), and PI (48.21±1.43% vs. 12.68±5.60%, *P* < .01). Moreover, left ventricular end-diastolic volume significantly increased (78.05±17.21 vs. 90.78±14.82 ml/m^2^, *P* < .01) after PVR. The other hemodynamics indexes did not change, significantly.

*Conclusion:* Despite the controversies about efficacy of PVR after repair of TOF, the remarkable improvement of hemodynamic is a supportive rationale for performing PVR surgery in TOF patients.

## Introduction

Tetralogy of fallot (TOF) is one of the most common congenital heart disease.^1^ Pulmonary insufficiency (PI) is a common adverse event after TOF repair. Chronic cardiac overload which caused by PI, can result in cardiac (RV) wall enlargement, RV failure, heart failure, arrhythmias and unfortunately sudden cardiac death. Pulmonary valve replacement (PVR) may be indicated for the purpose of preventing permanent alterations of cardiac walls and hemodynamics.^2,3^ Although PVR performed by numerous surgeons for many years, the effect of PVR still remains controversial. Despite the evidences of recovered cardiac function and hemodynamics post-PVR, the beneficial effect of PVR on prevention of alterations of ventricular mass, volumes, and hemodynamics remains unclear and indications for PVR are restricted to limited conditions such as RVOT dysfunction.^1^ In our center, symptomatic patients with the symptoms like as exercise intolerance, clinical arrhythmia, and right ventricular failure that unequivocally caused by severe PI and the asymptomatic patients with RVEDV greater than 170 ml/m^2^ in cardiac MRI (CMRI) were routinely undergoing PVR. This study was carried out with aim of assessment of the effect of PVR surgery on hemodynamic indexes of patients who undergone total repair of TOF.

## Material and Methods

### Patients and Study Design

After approval of institutional committee of ethic, 19 consecutive TOF patients who underwent PVR between July 2010 and October 2012 were included in our cohort. All patients was underwent TOF correction prior to PVR. Our study population included a number of 17 PVRs with bioprosthetic valves (using St. Jude Medical Epic^TM^) and 2 PVRs with mechanical valves (using St. Jude mechanical valves).

The PVRs performed on cardio-pulmonary pump (CPB) or on beating heart (in the absent of right to left shunt). The size of valve was selected based on surgeon’s preference (always one size larger than native valve annulus). Both of mechanical or bioprosthetic valves were inserted into orthotopic position.

All data of these PVRs was collected from review of medical records of surgeon and hospital database. Moreover, results of CMRI studies were collected during follow up clinical visits at the time between 6th and 12th month after PVR.

### Statistical Methods

Data were analyzed using SPSS software (version 16, SPSS Inc, Chicago, IL, USA). Categorical data described as frequency (percent) and continuous data presented as mean ± standard deviation. The student *t* test was used to find any significant difference between hemodynamic parameters before and after surgery**.** The *P* value less than 0.05 was considered as significant level for all tests.

## Result

The perioperative and late outcomes of patients after PVR surgery was summarized in Table 1. As the Table 1 shows, TOF with pulmonary stenosis (PS) is the most common (63.2%)diagnosis of our patients, and the rest of them (26.3%) had TOF with pulmonary atresia (PA), and TOF with PA and PS (10.5%). The sternum of five patients (26.3%) remains open as a strategy, because of cardiac failure, edema, and prolonged CPB. Total ICU stay of patients was 2.96±036 and their hospital stay was 15.00±1.36 day. Additionally, the only one death after PVR (with mechanical valve), was related to right ventricular failure which caused by thrombosis of prosthetic valve.

**Table 1:** Characteristics of Patients

**Variable**	**Mean±SD or No. (%)** ^a^
Prioperative	
Age, year	12.00±5.31
Gender	
Male	6 (31.6%)
Female	13 (68.4%)
Diagnosis	
TOF+PA	5 (26.3%)
TOF+PS	12 (63.2%)
TOF+PA+PS	2 (10.5%)
Open sternum strategy	5 (26.3%)
Renal failure	0 (0%)
Cerebral accident	1 (5.3%)
ICU stay, day	2.96±036
Hospital stay, day	15.00±1.36
Late outcomes ^b^	
Valve dysfunction	1 (5.3%)
RV failure	1 (5.3%)
Ventricular tachycardia	0 (0%)
Infection	0 (0%)
Thromboembolism	1 (5.3%)
Mortality	1 (5.3%)

Abbreviations‏: BPV, bioprosthesis valve; MCV, mechanical valve; TOF, tetralogy of fallot; PA, pulmonary atresia; PS, pulmonary stenosis; ICU, intensive care unit; RV, right ventricular. ^a^ Continues data are presented as Mean ± Standard Deviation and Categorical data are presented as Frequency (Percentage) ^b^ All of late outcomes are related to first year follow-up visits after PVR surgery.

As the Table 2 shows, right ventricular volumes significantly decreased after PVR surgery. The Right ventricular end-diastolic volume (RVEDV) shows significant decrease in follow-up measurements (180.89±13.78 vs. 107.21±12.02, *P* <.01). Also, the Right ventricular end-systolic volume (RVESV) significantly decreased after surgery (105.42±15.98 vs. 58.15±11.67, P<.01). Correspondingly, the CMRI results show the substantially shrinkage of right ventricular (RV) mass after PVR (47.78±6.20 vs. 30.68±8.95, P<.01). Moreover, right ventricular ejection fraction (RVEF) improved after valve replacement, but this improvement was not statistically significant (41.45±5.45 vs. 43.30±9.67, P=.11).

**Table 2:** Comparison of CMRI Characteristics of Patients Before and After PVR

**Variable** ^a^	**Subjects (n=19)**		*** P *** ** Value**
	**Before PVR**	**After PVR**	
RVEDV (ml/m^2^)	180.89±13.78	107.21±12.02	<0.01
RVESV (ml/m^2^)	105.42±15.98	58.15±11.67	<0.01
RV EF (%)	41.45±5.45	43.30±9.67	0.11
RV mass (g/m^2^)	47.78±6.20	30.68±8.95	<0.01
LVEDV (ml/m^2^)	78.05±17.21	90.78±14.82	<0.01
LVESV (ml/m^2^)	37.04±7.19	38.82±6.30	0.67
LV EF (%)	47.07±18.31	55.77±7.86	0.09
PI (%)	48.21±1.43	12.68±5.60	<0.01

Abbreviations: CMRI, cardiac magnetic resonance imaging; PVR, pulmonary valve replacement; RVEDV, right ventricular end-diastolic volume; RVESV, right ventricular end-systolic volume; RVEF, right ventricular ejection fraction; RV mass= right ventricular mass; LVEDV, left ventricular end-diastolic volume; LVESV, left ventricular end-systolic volume; LVEF, left ventricular ejection fraction; PI, pulmonary insufficiency. ^a^ Continues data are presented as Mean ± Standard Deviation ad analyzed using *t* test.

**Figure 1:**
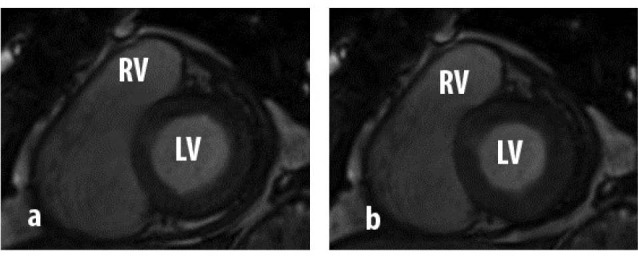
The CMRI image of an 18 year old male patient before and after PVR.

As depicted in Figure 1, the alterations of left ventricular volumes show advantageous effect of PVR on left ventricle. Our result shown that PVR increased quantities of Left ventricular end-diastolic volume (LVEDV) (78.05±17.21 vs. 90.78±14.82, P<.01), left ventricular end-systolic volume LVESV) (37.04±7.19 vs. 38.82±6.30, P=.09), and left ventricular ejection fraction (LVEF) (47.07±18.31 vs. 55.77±7.86, P=.09). As our result shown, although the improvement of LV hemodynamics, only increase of LVEDV was statistically significant. Furthermore, PVR surgery resulted in significant decrease in incidence of PI (48.21±1.43% vs. 12.68±5.60%, P<.01).

## Discussion

Chronic forces against the cardiomyocytes is a trigger for muscular hypertrophy and cardiac wall thickening.^4^ Therefore, correction of PI can result in shrinkage of RV mass by decrease of RV load. In accordance with previous studies, our results indicate a significant decrease in RVEDV and RVESV after PVR.^5,6^ These results confirm the hypothesis that RV mass can significantly decreased by PVR. In other words, PVR can gradually result in RV remodeling by removal of overloaded pressure on RV myocytes. Surprisingly, despite the shrinkage of expanded RV wall and improvement of RV hemodynamic, RVEF failed to show a significant improvement. Bove and colleagues^5^ founded a significant shrinkage of dilated RV (RVD) and improvement in exercise tolerance after PVR surgery but they reported only a trivial increase in RVEF. In contrary, the report of Vliegen et al^7^ indicated that RVD and RVEF can improve after the surgery.

Evidences suggest that the probability of RV regression considerably decreases in presence of preoperative RVEDV>160-170 ml/m^2^ or RVESV>80-90 ml/m^2^.^6^ Therefore, we concluded that the efficacy of PVR is limited to the RV volumes lower than this limit. Despite the fact that PVR can effectively reduce RV size, its effects on RV function may be limited because of the increased scar and fibrotic tissue in the RV wall.

Silberman et al^4^ studied the behavior of ventricles after implantation of stentless valves, by assessment of gradients and effective orifice area (EOA) index in patients who underwent aortic valve surgery. They concluded that rapid improvement of ventricular function is highly depended on valve performance rather than ventricular regression. High gradient across the functioning prosthetic valve can suspend recovery of RV function. Therefore, another probability is that improvement of RV function is a long-term process that may need more time to develop. The current evidences suggest that early restoration of pulmonary valve function can fully normalize the RV hemodynamic, but the exact time of PVR remains unknown.^8,9^

LV dysfunction frequently is a common finding in symptomatic patients with TOF and implies that the presence of the severe RV disease may be a cause for LV dysfunction. Therefore, management of congenital RV anomalies may improve the LV function and clinical symptoms.^10^ Likewise, our results show that the PVR surgery could effect on LV volumes as well as RV volumes. It was postulated that interaction between LV and RV chambers is mainly related to mutual myocardial fibers between LV and RV. Additionally, incline of the nestled interventricular septum into the LV cavity can decrease left ventricular refilling during diastole phase. Hence, LV hypertrophy (LVH) could result as an important compensatory mechanism for unfilled LV to increase cardiac output.^6,11^ Our study supports this experience and show that parameters of LVEDV (significantly), LVESV, and LVEF (non-significantly) increased after PVR.

This study has several limitations. There is a noticeable difficulty in homogeneous surgical recruitment of patients because patients may be referred for surgery at different stages of PI or RVOT dysfunction. Moreover, small size of our study population counts as another major limitation of this study.

## Conclusion

In conclusion, our study indicates that PVR surgery can successfully normalize the impaired hemodynamic of patients who previously underwent repair of TOF. Although there is a controversy about effect of PVR in TOF patients among experts, the remarkable improvement of hemodynamic after PVR is a supportive rationale of performance of this surgery before RV hemodynamic reaches to RVEDV>160-170 ml/m^2^ or RVESV>80-90 ml/m^2^.

Unexpectedly, despite the fact that PVR significantly improves RV size and volume, RV function remained impaired after PVR. We concluded that early PVR surgery can initiate RV remodeling and improvement of RV function. Hence, in order to prevention of development irreversible alterations in cardiac walls, the PVR surgery should be consider in TOF patients with the upper threshold levels as soon as possible.
